# Unlocking insights: text mining analysis on the health, welfare, and behavior of cows in automated milking systems

**DOI:** 10.1093/jas/skae159

**Published:** 2024-06-08

**Authors:** Giulia Gislon, Luciana Bava, Maddalena Zucali, Alberto Tamburini, Anna Sandrucci

**Affiliations:** Department of Agricultural and Environmental Sciences - Production, Territory, Agroenergy (DiSAA), University of Milan, 20133, Milan, Italy; Department of Agricultural and Environmental Sciences - Production, Territory, Agroenergy (DiSAA), University of Milan, 20133, Milan, Italy; Department of Agricultural and Environmental Sciences - Production, Territory, Agroenergy (DiSAA), University of Milan, 20133, Milan, Italy; Department of Agricultural and Environmental Sciences - Production, Territory, Agroenergy (DiSAA), University of Milan, 20133, Milan, Italy; Department of Agricultural and Environmental Sciences - Production, Territory, Agroenergy (DiSAA), University of Milan, 20133, Milan, Italy

**Keywords:** automatic milking system, behavior, health, text mining, topic analysis, welfare

## Abstract

Automated Milking Systems (AMS) have undergone significant evolution over the past 30 yr, and their adoption continues to increase, as evidenced by the growing scientific literature. These systems offer advantages such as a reduced milking workload and increased milk yield per cow. However, given concerns about the welfare of farmed animals, studying the effects of AMS on the health and welfare of animals becomes crucial for the overall sustainability of the dairy sector. In the last few years, some analysis conducted through text mining (TM) and topic analysis (TA) approaches have become increasingly widespread in the livestock sector. The aim of the study was to analyze the scientific literature on the impact of AMS on dairy cow health, welfare, and behavior: the paper aimed to produce a comprehensive analysis on this topic using TM and TA approaches. After a preprocessing phase, a dataset of 427 documents was analyzed. The abstracts of the selected papers were analyzed by TM and a TA using Software R 4.3.1. A Term Frequency-Inverse Document Frequency (TFIDF) technique was used to assign a relative weight to each term. According to the results of the TM, the ten most important terms, both words and roots, were feed, farm, teat, concentr, mastiti, group, SCC (somatic cell count), herd, lame and pasture. The 10 most important terms showed TFIDF values greater than 3.5, with feed showing a value of TFIDF of 5.43 and pasture of 3.66. Eight topics were selected with TA, namely: 1) Cow traffic and time budget, 2) Farm management, 3) Udder health, 4) Comparison with conventional milking, 5) Milk production, 6) Analysis of AMS data, 7) Disease detection, 8) Feeding management. Over the years, the focus of documents has shifted from cow traffic, udder health and cow feeding to the analysis of data recorded by the robot to monitor animal conditions and welfare and promptly identify the onset of stress or diseases. The analysis reveals the complex nature of the relationship between AMS and animal welfare, health, and behavior: on one hand, the robot offers interesting opportunities to safeguard animal welfare and health, especially for the possibility of early identification of anomalous conditions using sensors and data; on the other hand, it poses potential risks, which requires further investigations. TM offers an alternative approach to information retrieval in livestock science, especially when dealing with a substantial volume of documents.

## Teaser

According to the results of the text mining, the ten most important terms, both words and roots, were feed, farm, teat, concentr, mastitis, group, SCC (somatic cell count), herd, lame, and pasture.

Eight topics were selected with topic analysis, namely: 1) Cow traffic and time budget, 2) Farm management, 3) Udder health, 4) Comparison with conventional milking, 5) Milk production, 6) Analysis of AMS data, 7) Disease detection, and 8) Feeding management.

## Introduction

The first dairy cow was milked without traditional human involvement in 1986, using a robotic milking box at the Waiboerhoeve experimental farm in Lelystad (Netherlands) by Gascoigne Melotte, following the US Patent 4010714A (Notsuki & Ueno, 1977). The first milking robot on a commercial dairy farm was installed in 1992, in the Netherlands (John et al., 2016), and the first publication on the topic appeared in 1995. Since then, Automatic Milking Systems (AMS) have become more and more widespread in dairy farms: after 30 yr, the technical evolution has allowed these machines to achieve a very high level of reliability and efficiency, thus favoring their commercial spread. It is estimated that around 50,000 milking robots operate worldwide, of which over 1,200 are in Italy (4% to 5% of Italian dairy cattle farms) (Calcante, 2022).

The AMS on dairy farms offers two major advantages when compared to conventional milking: a reduction in the workload for milking and an increase in milk yield per cow (Rotz et al., 2003). Wage rates are increasing and sourcing high-quality milking labor is challenging. The profitability of AMS in comparison to the parlor systems depends on factors such as their economic life, attachment, and milking times (Salfer et al., 2017). If the AMS replaces the farmer work, it also provides time-saving, allowing farmers to allocate more time to farm management or family and leisure, thereby improving their quality of life.

Several studies have demonstrated that AMS leads to an increased milking frequency of up to three times a day, resulting in a 3% to 11% enhancement in milk yield compared to a conventional milking system which usually involves milking cows twice daily (Baines, 2002; De Koning, 2004; Hogenboom et al., 2019).

Farm management changes substantially after the introduction of AMS, as well as the animal-farmer relationship. Farmers have to rely on data recorded by the robot’s sensors to monitor the health status of the cows, rather than on visual observations during milking. Different brands of AMS record quite different sets of data, even though all of them provide data for monitoring animal health, welfare, and fertility. Data collected by AMS can offer valuable insights into the overall conditions of the animals. In the international literature, several studies focused on the use of data collected by the milking robots for animal health status detection, mainly concerning udder health (Berglund et al., 2002; Hovinen & Pyörälä, 2011; Zucali et al., 2021). Milking robots usually provide data on milk yield and flow per milking and per quarter. Some AMS use milk electrical conductivity and milk color for mastitis alert (Khatun et al., 2018; Inzaghi et al., 2021) while others, directly measure milk somatic cell count optically. Additional sensors in milking robots, such as 3D cameras for body condition scores, automatic systems for measuring weight or assessing lameness, can enhance cow welfare and health monitoring (Silva et al., 2021).

Although AMS provides ample data for monitoring cows’ health, there are limited studies in the international literature on the long-term effects of adopting robotic milking on the actual welfare and behavior of dairy cows, as well as human-animal relationships (e.g., Rodenburg, 2012). Schillings et al. (2021) examined the possible impacts of precision livestock farming (PLF) on animal welfare using the Five Domains Model. They concluded that although current PLF technologies broadly have the potential to reduce negative welfare issues, such as injuries or illness, they are not yet able to promote positive welfare. However, such limitations may not entirely be attributed to technology, as there is an active scientific inquiry into reliable indicators of positive welfare states, regardless of the approach used to detect such indicators in animals. Dawkins (2021) stated that the extent to which PLF improves the welfare of livestock on commercial farms depends on the definition and consensus regarding welfare among the various human actors involved in developing and using the technology, as well as the public. Recently, Tuyttens et al. (2022) discussed the potential threats of PLF technologies to animal welfare identifying 12 potential threats, grouped into four categories: direct harm, indirect harm, changes to housing and management, and ethical stagnation or degradation.

While the spread of technology in commercial farms has increased, there is also a growing public concern for the health and welfare issues of livestock. Consumers and citizens, indeed, show a great interest in the environmental impact associated with both human activities and animal welfare. They ask for animal products healthy and of high organoleptic quality, obtained through rearing techniques that are environmentally friendly and respectful of animal welfare (Cembalo et al., 2016). Furthermore, they are willing to pay more for food produced using sustainable methods (Canavari and Coderoni, 2020).

Since the health and welfare of farmed animals are considered fundamental both in themselves and for the environmental, social, and economic sustainability of animal production (Buller et al., 2018), it follows that, if robotic milking systems were proven to be incapable of ensuring high standards of animal conditions, they would be excluded from the race for sustainability.

In the last few years, analysis in the scientific literature conducted through text mining (TM) and topic analysis (TA) approaches has become increasingly widespread (e.g., Nalon et al., 2021; Zuliani et al., 2021). Considering the livestock sector around 20 analyses using TM and TA approaches have been published to date.

The TM and TA techniques represent tools that can produce a preliminary thematic screening of large volumes of documents to reveal a structured map of textual knowledge, by uncovering recurrent topics and latent themes when the set of documents to analyze is large (Wang et al., 2016; Park & Kremer, 2017). In particular, TM represents a knowledge discovery process, looking for extracting valuable information from vast amounts of textual data. This technique can analyze concept relationships, to find new structures, patterns, or associations and to uncover novel facts and trends about the world itself (Abutridy, 2000). Therefore, TM can be used to summarize information into charts or maps, identify hidden structures between concepts or groups or concepts, extract hidden associations between textual elements, provide an overview of the contents of a large collection of documents, and categorize texts by discovering relevant groupings (Abutridy, 2000).

Although other scientific papers have been published on the use of AMS, there are no recent studies specifically focused on the health, welfare, and behavior of dairy cows performed with TM and TA (Jacobs and Siegford, 2012; Cogato et al., 2021).

The aim of the study was to analyze the international scientific literature on the use of AMS and its impact on dairy cow health, welfare, and behavior: the paper aimed to produce a comprehensive investigation on this topic using TM and TA approaches.

## Materials and Methods

### Database preparation

**Figure 1.**
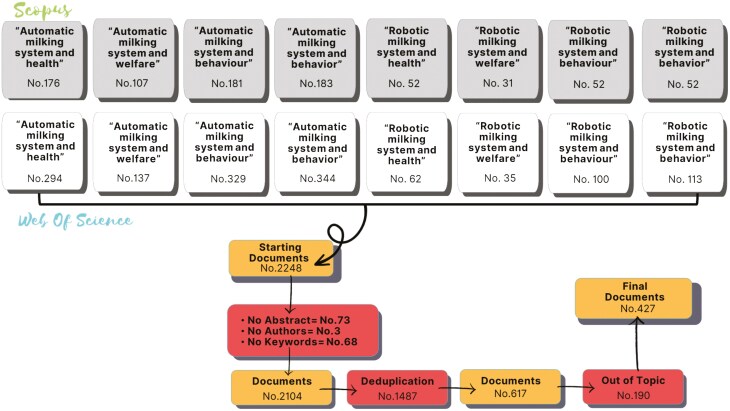
Final dataset construction. Shaded boxes are referred to Scopus documents and non-shaded boxes are referred to Web of Science documents.

The database of abstracts that covered the topic of the present study was created by searching documents in Scopus and Web of Science (WOS) scientific databases. The search strings used were: “Automatic milking system and health,” “Automatic milking system and welfare,” “Automatic milking system and behaviour” “Automatic milking system and behavior,” “Robotic milking system and health,” “Robotic milking system and welfare,” “Robotic milking system and behaviour,” and “Robotic milking system and behavior”. The term “behaviour/behavior” was therefore searched using both English and American spelling. For Scopus it was adopted the restriction criterion of Subject Area is Limited to: Agricultural and Biological Sciences, Veterinary and Environmental Science. For WOS the Subject Area was limited to: Agriculture Dairy Animal Science, Food Science Technology, Veterinary Sciences, and Environmental Sciences. No restriction concerning the year of publication and document type was adopted. Therefore, the documents selected were original articles, reviews, book chapters and conference papers. We restricted the selected publications to those having abstracts written in the English language. A total of 2,248 documents were extracted. Reasons for subsequent exclusion from the database were: no author available, no abstract available, and no keywords available. In addition, a screening of all the articles was performed to delete the duplicated ones. Additionally, two of the authors manually screened the database to eliminate the abstract considered out of the topic. The final dataset consisted of 427 documents. The construction of the final dataset took place in July 2023: a detailed description of the process involving the dataset construction is shown in Figure 1.

### Descriptive statistics

Some descriptive statistics of the database, based on information recorded from Scopus and WOS, were performed to describe the literature selected. Particularly, the distribution analyses by year and by journal of the published documents were performed, to explore the trend of the scientific interest for this topic. All statistics were made with the Software R 4.3.1.

Vosviewer (https://www.vosviewer.com/) was used to analyze the research hot spots. By extracting the titles and abstracts of the documents, the research hot spots were visualized in the form of a network. For this analysis, no database preprocessing was done (e.g., stemming, word length ≥ 3, remotion of the keywords used in the literature search). The bubble size represented the word frequency, and the curves represented the links between words.

### Text mining

A TM analysis was performed on the abstracts of the selected papers to find the most important words of the data *corpus* and their associations. The TM approach converts the text into a numeric information and analyzes the word frequency distributions (Nalon et al., 2021; Zuliani et al., 2021). For TM the Software R 4.3.1. was used, and the R library “tm” was used. To preprocess the text data, words were converted to lowercase and any unusual characters were removed. Punctuation, common English stopwords, numbers, and extra white spaces were also eliminated. In addition, keywords used in the literature search were removed to avoid low discriminative information due to their presence in almost all retrieved abstracts (Wang et al., 2016; Nalon et al., 2021). Finally, stemming was performed to reduce all words to their root form using the R library “SnowballC”.

A document term matrix (DTM) was created to organize the terms (both terms and roots) into a matrix with the documents in the rows and the terms in the columns. A Term Frequency-Inverse Document Frequency (TFIDF) technique was used to assign a relative weight to each word (Salton & Buckley, 1988). This represents how important a word is in the whole collection of documents. Only terms with a length ≥ 3 were kept for the DTM, to eliminate dull terms, but keeping important words for our analysis e.g., the word “SCC (somatic cell count)”. Terms that were scattered were removed from the matrix, reducing the DTM from 4151 to 1265 terms. A graphical representation of the most important terms (by keeping only those with a weight ≥ 3, for highlighting in a graphical way frequencies of the most important terms only) was created with the R library “ggplot2”, and the terms were represented as a histogram. The association between terms with frequencies ≥ 3 was evaluated by considering a correlation limit of 0.4. Different trials were performed with different thresholds of both minimum length and frequencies, to identify the final thresholds, to highlight the relevant results, and in accordance with the international bibliography (e.g., Nalon et al., 2021).

Bigrams and trigrams analysis were also performed, by using R library “RWeka”, and the first 10 bigrams and trigrams were plotted on the weight basis.

### Topic analysis

Topic modeling analysis was performed using the Software R 4.3.1. for clustering terms and documents in the *corpus*, into homogeneous groups based on the research topics. R libraries “topicmodels,” “tidytext,” and “dplyr” were used.

This analysis is useful to uncover the structure of meaningful themes among collections of documents as well as to discover hidden textual patterns (Zuliani et al., 2021).

The approach of Latent Dirichlet Allocation (Blei et al., 2003) was used for topic modeling. Typical values of alpha and beta were used, that is 50/k (with k being the number of topics) and 200/W (with W being the number of words in vocabulary), respectively (Griffiths and Steyvers, 2004).

Each term retained by the TM was scored inside each topic based on the probability of belonging to that specific topic (beta). Terms are typically sorted in descending order of probability. The first 10 terms for each topic were chosen as those that most characterize the topic.

The number of topics (k) was selected to provide a comprehensive description of the *corpus* and a percentage of documents per topic ≥ 5%. Different trials were performed, namely 5, 8, 10, 15, and 20 topics; eight topics were eventually selected.

Each topic was labeled based on the most important terms and documents that belonged to it.

Graphical representation of the TA was performed by using “ggplot2” R library.

Statistical analysis for the topic trend over the years was performed using Statistical Analysis Software (SAS) software by Proc freq and Proc reg models. Data for 2023 were also reported, even though the year was not over yet. The chart was made using the Software R 4.3.1.

## Results

### Descriptive statistics

The final number of documents included in the study was 427 (Figure 1).

**Figure 2.**
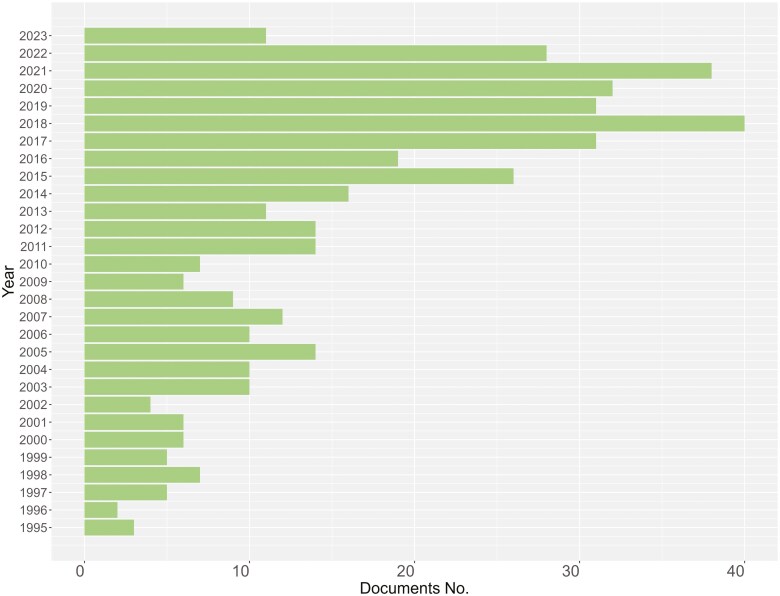
Distribution of published documents on automatic milking systems and cow health, welfare, and behavior by year.

The distribution of published documents by year is shown in Figure 2. Most of the documents were published in 2018 (No. 40), although research on these subjects started in 1996, with two documents published. An increasing number of documents were then published until 2018, after which there was a slight decline (Figure 2).

**Figure 3.**
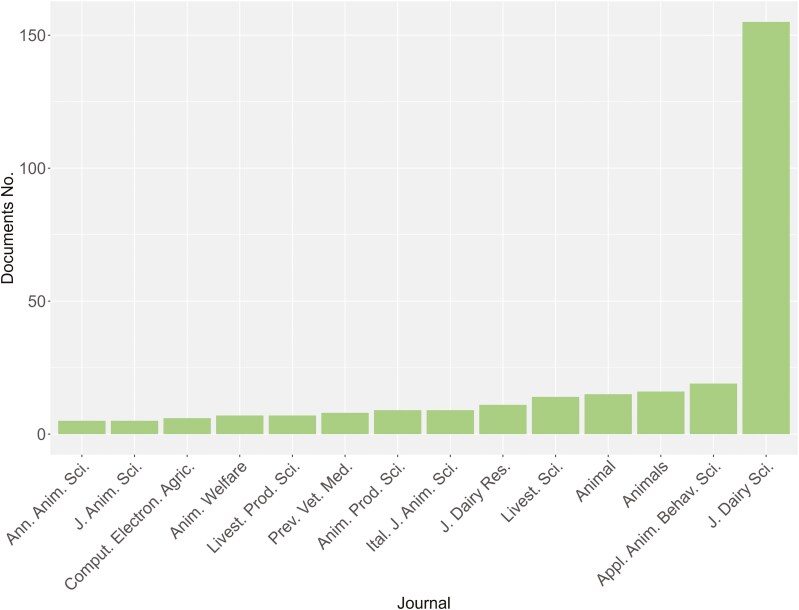
Distribution of published papers on automatic milking systems and cow health, welfare, and behavior by journal, considering only journals with number of publications ≥ 5.

Looking at the trend in the number of papers per journal (Figure 3), out of 105 sources that contained documents (including journals, conference proceedings, and books), only 14 collected more than five documents, with the Journal of Dairy Science (JDS) having the highest number of papers (155). A large difference was observed between JDS (155 papers) and the other sources (average value of 10.1 ± 4.55 documents). Among all the sources, eight published less than 10 documents and none of them, except JDS, published at least 20 documents. Most of the published documents were journal articles; in fact, all the sources with number of publications ≥ 5 were scientific journals. Almost all the journals were open-access, except for *Livestock Production Science* which was a hybrid journal.

Four of the journals considered are from England, and the other four are from the Netherlands. One journal is from Italy (*Italian Journal of Animal Science*; Figure 3). Considering the journal Impact Factor (IF) for 2022, provided by Journal Citation Report (2023), 8 journals have an IF ≥ 2, with the highest for *Computers and Electronics in Agriculture* (IF 2022 of 8.3). In terms of classification in quartile (Q) ranking, that is based on the IF of the journals in the same subject category provided by JCR, half of the journals considered in Figure 3 are in Q1 for AGRICULTURE, DAIRY & ANIMAL SCIENCE, excepting for *Preventive Veterinary Medicine* that is classified in Q1 for VETERINARY SCIENCES and *Computers and Electronics in Agriculture* that is classified in Q1 for AGRICULTURE, MULTIDISCIPLINARY. *Animal Welfare* (for VETERINARY SCIENCE) and *Animal Production Science* are in Q3, while all the other journals are in Q2.

**Figure 4.**
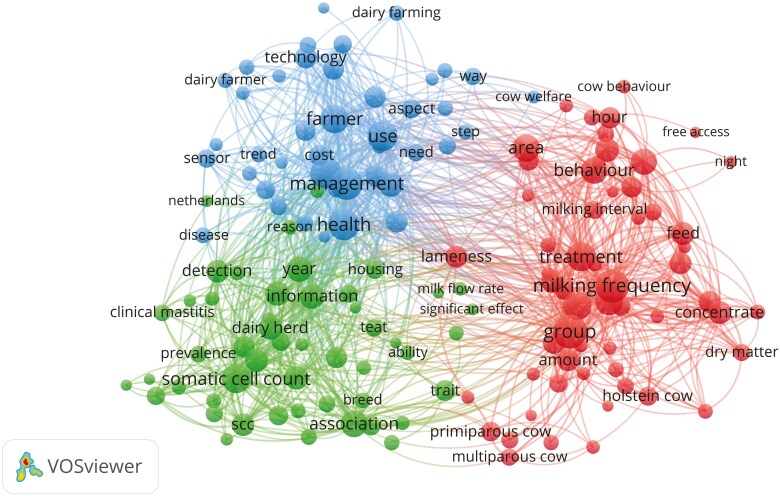
Research hot spots of automatic milking systems and dairy cow health, welfare, and behavior. The bubble size represents the word frequency, and the curves represent the links between terms.

Figure 4 shows the hot spots of international research on the topic, grouped into three categories represented by different colors. The first category (blue) is mainly related to aspect of farm “management” and the use of “technology” by farmers; in this category, the word “Health” carries significant weight and is closely related to management. The terms “somatic cell count,” “teat,” “detection,” and “clinical mastitis” in the second category (green) indicate a research focus on udder health issues related to the use of AMS, but also, conversely, on the possibilities offered by these systems to early detect mastitis problems. In the third category (red), the terms “group,” “milking frequency,” “concentrate,” “amount,” and “dry matter” suggest the focus of scientific papers on animal feeding management. The attention extends to the management of groups and cow traffic, milking intervals, and their implications on cow behavior.

### Text mining

**Figure 5.**
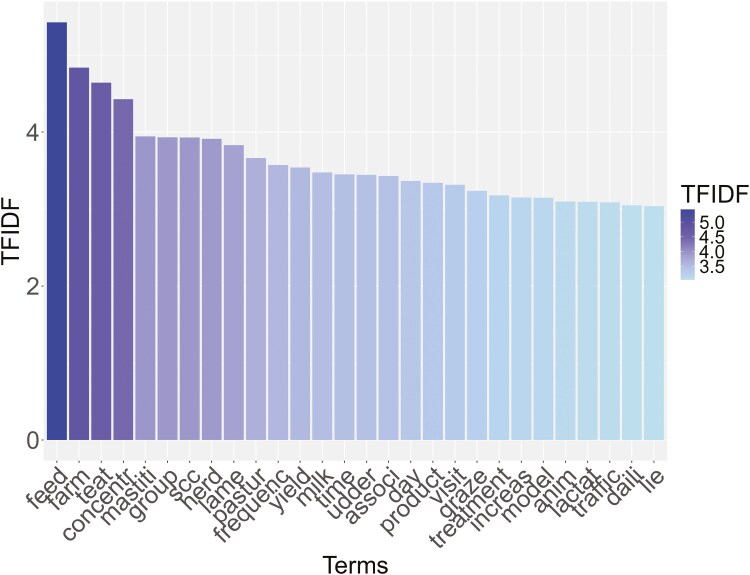
Graphical representation of the heaviest terms based on the Term Frequency-Inverse Document Frequency (TFIDF) by keeping only those with a TFIDF ≥ 3. Darker color means heavier terms.

The DTM consisted of 1,265 tokens, 996 bigrams, and 138 trigrams, and the most important terms (both terms and roots) according to the TFIDF ponderation system (TFIDF ≥ 3) are shown in Figure 5, in which each word is represented as a bar of the histogram.

Looking at Figure 5, the ten most important terms were feed (TFIDF = 5.43), farm (TFIDF = 4.84), teat (TFIDF = 4.64), concentr (TFIDF = 4.43), mastiti (TFIDF = 3.94), group (TFIDF = 3.93), SCC (somatic cell count) (TFIDF = 3.93), herd (TFIDF = 3.91), lame (TFIDF = 3.83), and pasture (TFIDF = 3.66).

Some interesting associations were found between the most frequent terms (considering only terms with frequencies ≥ 3 and a correlation (*r*) limit of 0.4), meaning that some terms were frequently found together in the documents. Some of the strongest associations were: “mastitis” associated with “alert” (*r* = 0.42), “model” associated with “decisionmak” (*r* = 0.42), “teat” associated with “clean” (*r* = 0.58), with “cup” (*r* = 0.57), with “attach” (*r* = 0.44), with “skin” (*r* = 0.41) and with “technic” (*r* = 0.41). Other interesting associations were found between “time” and “spent” (*r* = 0.51) and between “udder” and “conform” (*r* = 0.42). The word “traffic” resulted in being correlated with several terms, i.e., “free” (*r* = 0.68), “oneway” (*r* = 0.59), “forc” (*r* = 0.57), “rout” (*r* = 0.54), “area” (*r* = 0.43), and “cow” (*r* = 0.42).

In Table 1 results of bigram and trigram analysis are shown. Regarding bigram analysis, the ten most important bigrams were “milk yield,” “dairi cow,” “milk product,” “cow milk,” “dairi farm,” “cow traffic,” “somat cell,” “cell count,” “daili milk,” and “milk flow.”

**Table 1.** Results of bigram and trigram analysis

Bigram analysis		Trigram analysis	
Bigram	Weight	Trigram	Weight
Milk yield	426	somat cell count	100
Dairi cow	209	daili milk yield	60
Milk product	199	cell count scc	38
Cow milk	158	milk yield cow	31
Dairi farm	136	milk flow rate	28
Cow traffic	119	milk yield milk	27
Somat cell	117	free cow traffic	26
Cell count	110	dri matter intak	25
Daili milk	83	milk product cow	19
Milk flow	65	increas milk yield	18

The most important trigrams were “somat cell count,” “daili milk yield,” “cell count scc,” “milk yield cow,” “milk flow rate,” “milk yield milk,” “free cow traffic,” “dri matter intak,” “milk product cow,” and “increas milk yield.”

### Topic analysis

**Figure 6.**
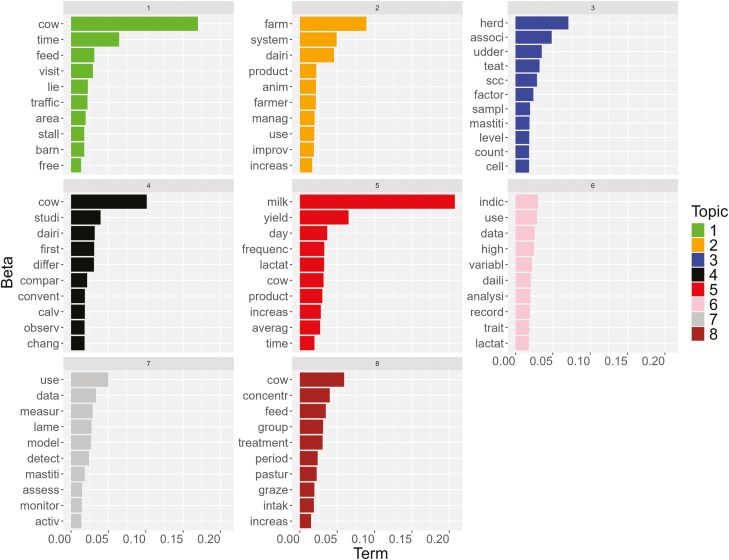
Most important terms per topic on automatic milking systems and cow health, welfare, and behavior from the Latent Dirichlet Allocation (LDA) with eight topics (beta = probability that a term belongs to a given topic).

The ten most probable terms in each of the eight identified topics are reported in Figure 6.

Based on the most important terms and the papers belonging to each topic, the authors attributed a label to each topic (Table 2).

**Table 2.** Topics that emerged on automatic milking systems and cow health, welfare, and behavior from the latent dirichlet allocation (LDA) analysis

Eight topic	Topic label	10 most probable terms	Documents no.	Documents %	First year publication	Cumulative probability	*P*-value	R^2^	Regression coefficient
**Topic 1**	Cow traffic and time budget	cow, time, feed, visit, lie, traffic, area, stall, barn, free	63	15	1995	0.41	< 0.0001	0.59	0.21
**Topic 2**	Farm management	farm, system, dairi, product, anim, farmer, manag, use, improv, increas	78	18	1997	0.32	0.002	0.31	0.16
**Topic 3**	Udder health	herd, associ, udder, teat, scc, factor, sampl, mastiti, level, count	50	12	1995	0.31	0.001	0.36	0.14
**Topic 4**	Comparison with conventional milking	cow, studi, dairi, first, differ, compar, calv, convent, chang, observ	37	9	2001	0.33	< 0.0001	0.49	0.16
**Topic 5**	Milk production	milk, yield, day, frequenc, lactat, cow, product, increas, averag, time	60	14	2000	0.51	0.0002	0.30	0.13
**Topic 6**	Analysis of AMS data	indic, use, data, high, variabl, daili, analysi, record, trait, lactat	31	7	2006	0.23	0.0002	0.40	0.10
**Topic 7**	Disease detection	use, data, measur, lame, model, detect, mastiti, assess, monitor, activ	55	13	2003	0.25	0.0003	0.39	0.16
**Topic 8**	Feeding management	cow, concentr, feed, group, treatment, period, pastur, graze, intak, increas	53	12	1996	0.29	0.89	0.0008	-0.005

From the results of TA emerged that the first year of publication on the use of AMS related to dairy cow health, welfare, and behavior was 1995, and publications belonged to topic 1 (Cow traffic and time budget) and topic 3 (Udder health). Topic 6 (Analysis of AMS data) was the last one that appeared in the international literature, published for the first time in 2006 (Table 2).

Topic 2 (farm management) collected the highest number and percentage of articles (18%). Topic 4 (comparison with conventional milking) and 6 (analysis of AMS data) had the lowest percentage of documents, less than 10%, while all the other documents were equally distributed among all the other topics (Table 2).

The analysis of cumulative probability statistically identified the two most important topics, namely topic 5 (milk production; cumulative beta 0.51) and topic 1 (cow traffic and time budget; cumulative beta 0.41).

The regression coefficient was positive for all topics except topic 8 (Feeding management), indicating an increasing number of documents for almost all topics throughout the year, with the highest increase for topic 1 and the smallest for topic 6.

**Figure 7.**
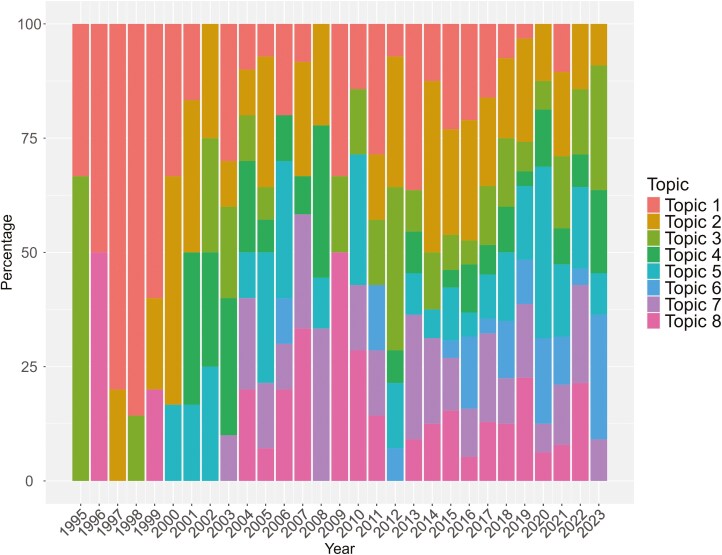
Trends in topics on automatic milking systems and cow health, welfare, and behavior over the years are expressed as percentages of the total papers on the subject published in the respective year.

Regression was significant for all the topics, except for the negative one, i.e., Topic 8 (Table 2). However, the overall trend of all the topics considered together resulted to be positive over the years (regression coefficient = 1.04; Pr > F < 0.0001; *R*^2^ = 0.65). Therefore, trends in topics may be better explained by the percentage of articles related to the different topics over the years (Figure 7).

As shown in Figure 7, during the first years of publication, only two topics were investigated, namely Topic 1 (Cow traffic and time budget) and topic 3 (Udder health); more and more subjects have been taken into account, during the years. During the first years, the most investigated topic was the one related to cow traffic and time budget (topic 1), even if in the first year of publication the most investigated one was topic 3 (Udder health). Considering the last years of publications (2020, 2021, and 2022) the most investigated topics were 5 (Milk production) and 6 (Analysis of AMS data).

## Discussion

The distribution of published documents over the years on animal health, welfare, and behavior with AMS underlined the growing interest within the academic community in these three macro thematic areas, along with the increasing spread of AMS on farms.

As highlighted by the analysis of the research hotspots, the three major macro thematic areas are the management of the technology by farmers, udder health, and feeding management. It is worth noting that, while the terms “health” and “behavior” bear considerable significance and occupy a central position in numerous connections, the term “cow welfare” exhibits a diminished impact and seems somewhat peripheral.

The most important term that resulted from the TFIDF ponderation system was “feed”, and one of the trigrams with the highest weigh was “dri matter intak”. The aspects of animal nutrition and feeding following the introduction of AMS in the farms, indeed, are considered crucial by the scientists. Usually, in conventional milking systems, cows receive all their nutrients from a total mixed ration; however, in herds equipped with AMS, a portion of their nutrients is provided during milking as a concentrate in the auto-feeder, mainly to attract cows to the milking system, whereas the remaining fraction is supplied in the feed bunk through a partial mixed ration or by utilizing pastures. Consequently, AMS presents both challenges and opportunities for feeding cows (Bach & Cabrera, 2017), with implications for animal health and welfare. On one hand, animals can be fed more closely to their needs with an individual-based concentrate feed delivery. On the other hand, the intake of significant quantities of starch-rich concentrates during robot visits, can negatively impact the course of ruminal fermentations, leading to alterations in pH (Bach & Cabrera, 2017). Three of the other important terms were “concentr,” “group,” and “pastur.” They appear among the most likely terms in topic 8 (Feeding management). This topic was one of the first subjects studied by researchers, with the first articles being published in 1996. The first AMSs were installed in Northern Europe, specifically in the Netherlands and Denmark (Barkema at al., 2015), where the use of pasture in dairy cow breeding is common. In recent years, publications on this specific topic have decreased, as evidenced by the negative regression, probably due to the broader adoption of AMS in various livestock systems with indoor management.

The terms “teat,” “mastitis,” “scc,” “udder,” “herd,” and “associ” were also classified as heavy by the TFIDF ponderation system, and this can be interpreted in two ways in relation to health, welfare, and behavior. In addition, bigrams “Somatic cell,” “cell count,” and trigrams “somat cell count,” and “cell count scc” in the same way were classified as heavy by the bigram and trigram analysis. These terms are referred both to the literature investigating opportunities for automated mastitis detection in AMS without human involvement (Zucali et al., 2021) and to the research on the effect of AMS on the risk of mastitis development (Hamann & Reinecke, 2002). During the first years following the spread of AMS, topic 3 (“Udder health”) was extensively investigated, with those kinds of publications beginning in 1995. The milking robot may be considered one of the earliest developments in PLF that has revolutionized dairy farming worldwide (John et al., 2016). The most innovative aspect is the absence of a human milker, along with an increasing number of milkings per day. These two aspects likely initially oriented research toward the study of the impact of AMS on the risk of mastitis in herds equipped with AMS as the absence of a human could suggest less monitoring of udder health, while increased milk production could heighten animals’ susceptibility to mammary infections. In more recent studies, robotic milking is seen as an opportunity to early diagnose changes in udder health status through the exploitation of data collected by the AMS at each milking. Topic 6 (Use of data provided by the robot), indeed, was the most recent one, and one of the topics covering the largest percentage of documents in recent years. The analyzed international literature highlights that, with an average number of daily milkings up to 3, at herd level, AMS enables farmers to collect a huge amount of real-time data on individual cows or at a quarter level (milk production, flows, conductivity, etc.), allowing for a level of detail in monitoring never achieved before. The big data generated by the multiple sensors implemented in AMS can be statistically managed for the development of algorithms for the real-time detection of changes enabling the farmer to make quick and informed decisions for enhancing animal health and welfare (Zucali et al., 2021).

The term “Lame” emerged as one of the most important in the international scientific literature related to AMS and dairy cow health, welfare, and behavior. In the early 2000s, increased attention was directed towards disease detection (topic 7), particularly lameness, resulting in the publication of numerous studies beginning from that period. Together with mastitis, lameness is currently one of the main health concerns facing the livestock sector, particularly in the dairy cattle industry (Afonso et al., 2020). Lameness arising from foot or claw lesions is one of the most painful conditions in dairy cattle (Whay and Shearer, 2017). The impact of lameness in dairy cows is a reduction in animal welfare, reproductive performances (Garbarino et al., 2004), and economic efficiency (Ózsvári, 2017). The health of the hoof and normal mobility becomes essential in herds milked automatically to facilitate the cow’s access to the robot and, in the presence of guided traffic, to the feed bunk (Miguel-Pacheco et al., 2014). Therefore, AMS can have an additional negative effect on the welfare of lame animals due to the adverse impact of delayed milking on udder health and potential reduction in feed intake. The presence of lameness increases the number of alarms for cows that are late for milking, necessitating numerous direct fetching interventions by the farmer to encourage the animals to go to the robot.

“Farm” was the second important word related to dairy cows’ health and behavior from the *corpus* of analyzed documents. Transitioning from a conventional milking system to an AMS, as mentioned, requires a new management approach (Svennersten-Sjaunja & Pettersson, 2008), encompassing several aspects including change in labor tasks, as well as adjustments in the herd management and group dynamics. The analyzed literature reported that transitioning from a milking parlor to an AMS may lead to significant changes for both herdsman and the cow, potentially causing stress to both parties. Studies on the new farm management practices (topic 2) began to spread from the first years when AMS were installed, starting from 1997. The installation of milking robots, each managing 60 to 70 cows, implies voluntary milking which, on one hand, represents an increase in freedom for the animals. However, as already mentioned, it can sometimes lead to delays in milking for cows with mobility difficulties, or lazy animals. On the other hand, the groups of animals are rather large, which can trigger competitive behavior and aggressive interactions at the entrance of the robot or at the feeding trough with negative effects on the welfare of cows, especially the subordinate ones (Danielsson, 2012). Indeed, “group” was the sixth most important word from the TM analysis.

Dairy cows are social animals, therefore positive social interactions between animals may also occur with AMS. As reported by Fadul-Pacheco et al. (2021), there are affinity pairs that are formed and broken as cows entered and left the social group (pen). These cows’ social relationships impact the day-to-day variability in milk production as well as daily average milk production. AMS allows individual feeding through concentrate distribution in the robot. This reduces the need to move animals from one group to another during lactation, maintaining more consistent the group composition. Keeping the cow group constant promotes the maintenance of affinity pairs, leading to advantages for animal welfare.

With AMS, the milking process no longer requires permanent supervision. However, this does not necessarily imply a reduction in the time spent on milking. New labor tasks arise from the implementation of automatic milking in the farm: monitoring and cleaning of the AMS, checking alert lists twice or three times a day, visually inspecting the cows, and fetching cows that have exceeded maximum milking intervals (De Koning, 2004).

The new farm management also involves choosing the type of traffic: free, forced, or guided. Some of the most frequent terms showed strong associations, meaning that they are frequently found together in the documents. Among the word correlations, the most interesting ones were between “traffic” and the terms “free,” “oneway,” “forc,” “rout,” “area,” and “cow,” which probably trace a tendency in studies towards this aspect of herd management with AMS. This was also confirmed by bigram analysis, reporting “cow traffic” as one of the 10 most weighted bigrams, and even more by the trigram analysis, showing in the heavier words the trigram “free cow traffic”. Topic 1 (Cow traffic and time budget) is the one in which these terms appear most frequently. When installing a robotic milking system, selecting cow traffic becomes a focal point for farmers, therefore since 1995, several studies focused on this aspect. Free cow traffic allows cows to decide whether to visit the AMS or not, as opposed to forced cow traffic, where the AMS is the only route from the lying area to the feeding zone (Ketelaar-De Lauwere et al., 1998). According to the literature, forced cow traffic may improve the frequency of visits to the AMS, but somewhat alters the cows’ behavior, and may, therefore, be questionable. Free cow traffic could work if cows are previously conditioned to follow the route to the AMS (Ketelaar-De Lauwere et al., 1998). However, the issue of fetching the non-compliant cows into the robot often remains. In addition, many authors have stated that dominant animals in a cattle herd have priority whenever there is a competitive situation at the feeding site (McPhee et al., 1964; Friend and Polan, 1974; Kabuga, 1992). Ketelaar-De Lauwere et al. (1996) suggested that effects of social dominance will appear when AMS are introduced and that these effects may be seen in the timing of the visits to the AMS and to the feeding gate and the time spent in the waiting area in front of the AMS. Cows with low dominance values adapt their visits to the AMS and the feeding gate to the cows with higher dominance values by visiting both parts of the cowshed at quieter times. Cows with lower dominance values spend more time waiting in front of the milking robot. Therefore, the waiting area should be designed to prevent them from being subjected to severe aggression from higher-ranking cows. “Traffic and time budget,” along with “Udder health,” represents the first topic published on animal welfare and health related to robotic milking. Topic 1 was also the second most significant topic, statistically. Today it is well known that the ideal situation would be free traffic and nutritional approaches that would reduce variation in the number of visits to the AMS (Bach & Cabrera, 2017). However, the number of daily visits per cow to the AMS is also dependent on many other factors, such as milk production, stage of lactation, parity, group size and composition, or even the localization of the robot in the barn.

The comparison with milking parlor (Topic 4) was investigated since 2001. The international literature highlighted that even if, in the presence of AMS, farmers overall spent less time interacting with cows, the animals had become quieter in the AMS. Results from this study indicated that the farmer-cow relationship had improved after AMS transition and that this may be related to changes in farmer working routine (Wildridge et al., 2020).

The most important topic statistically was Topic 5 - “Milk production” and the terms “yield,” “milk,” “product,” “day,” “time,” and “frequenc” resulted as important. This result was not surprising since milk production is an important point of difference between AMS and conventional milking parlor, since the first generally allow cows to be milked up to three times a day, which increases milk production from 3% to 11%, compared to a conventional milking system (in which usually cows are milked twice daily) (Baines, 2002).

This was also confirmed by bigram analysis, revealing the importance given from the international bibliography to the milk production (“milk yield,” “dairi cows,” “milk product,” “cow milk,” “dairy farm,” “daily milk,” and “milk flow”). Trigram analysis also highlighted the high importance given to the animal productivity, trigrams such as “daily milk yield”, “milk yield cow”, “milk flow rate”, and “increase milk yield” resulted as heavy in the trigrams analysis.

One of the largest areas of potential improvement in the use of AMS appears to be using milking management strategies to increase the percentage of time that each AMS box is actually milking cows. This could be achieved by an optimal combination of milking permissions, udder fill limits, number of cows per AMS, with improved barn design and cow traffic strategies required to realize this potential (Reinemann et al., 2019).

The recurring themes in this group of papers were somatic cells and udder health connected with intervals in milking since cows milked with robots usually go voluntarily to be milked (Nogalski et al., 2011; Ferneborg and Svennersten-Sjaunja, 2015). On the one hand, the robot offers interesting opportunities to safeguard animal welfare and health, particularly in terms of early identification of anomalous conditions. Moreover, voluntary milking provides a form of freedom for the animals allowing them to be milked at their preference. On the other hand, it poses potential risks, especially in relation to delayed milkings, which can reduce productivity and induce udder issues in lame, lazy, or subordinate cows. The problem of fetching delayed cows is not entirely resolved even with the distribution of concentrated feed in the robot to attract animals. Considering the social and hierarchical aspects of the herd, the AMS can represent both an opportunity or a risk mainly in relation to the composition and dynamics of cow groups.

Overall, bigram and trigram analysis revealed that research related to health and welfare with AMS may be referred to three main areas by importance, i.e., animal productivity, udder health, and cow traffic.

## Conclusions

TM can be considered a powerful tool for bibliographic analysis, revealing a growing interest in the effects of automated milking on cow health and behavior within the research community, with an evolution of the research focus from traditional aspects like cow traffic and udder health to data analysis and monitoring. What appears to emerge from the analysis is still the complex nature of the relationship between automated milking and animal welfare and health, particularly regarding feeding management, grouping and traffic, and the most common diseases, such as mastitis and laminitis.

Stakeholders can use this insight to prioritize data-driven approaches for monitoring animal conditions and promptly identifying signs of stress or disease.

Researchers need to further investigate the topic to address public concerns about animal welfare and ensure the sustainability of the dairy sector. Prioritizing transparency and communication efforts is crucial to bridge the gap between research findings and public perception, while also implementing practices that promote the well-being of animals and the dairy industry.

## Abbreviations

AMS: automated milking systems
DTM: document term matrix
IF: impact factor
JDS: Journal of Dairy Science
PLF: precision livestock farming
pH: potential of hydrogen
Q: quartile
SAS: statistical analysis software
SCC: somatic cell count
TM: text mining
TA: topic analysis
TFIDF: term frequency-inverse document frequency
WOS: Web of Science
